# High-Altitude Hypoxia Induces Excessive Erythrocytosis in Mice via Upregulation of the Intestinal *HIF2a*/Iron-Metabolism Pathway

**DOI:** 10.3390/biomedicines11112992

**Published:** 2023-11-07

**Authors:** Sisi Zhou, Jun Yan, Kang Song, Ri-Li Ge

**Affiliations:** 1 Research Center for High Altitude Medicine, Qinghai University, Xining 810001, China; sisizhouqhdx@outlook.com (S.Z.); Yjqhdx@163.com (J.Y.); Skqhsrmyy@163.com (K.S.); 2 Key Laboratory of High-Altitude Medicine, Ministry of Education, Xining 810001, China; 3 Key Laboratory of Application and Foundation for High Altitude Medicine Research in Qinghai Province, Xining 810001, China

**Keywords:** excessive erythrocytosis, iron metabolism, intestinal *HIF2α*, high-altitude hypoxia, hypoxia-inducible factor 2 alpha

## Abstract

Excessive erythrocytosis (EE) is a preclinical form of chronic mountain sickness (CMS). The dysregulation of iron metabolism in high-altitude hypoxia may induce EE. The intestinal hypoxia-inducible factor 2 alpha (*HIF2a*) regulates the genes involved in iron metabolism. Considering these findings, we aimed to investigate the function and mechanism of intestinal *HIF2α* and the iron metabolism pathway in high-altitude EE mice. C57BL/6J mice were randomized into four groups: the low-altitude group, the high-altitude group, the high-altitude + *HIF2α* inhibitor group, and the high-altitude + vehicle group. In-vitro experiments were performed using the human intestinal cell line HCT116 cultured under hypoxic conditions for 24 h. Results showed that high-altitude hypoxia significantly increased the expression of intestinal *HIF2α* and iron metabolism-related genes, including *Dmt1*, *Dcytb*, *Fpn*, *Tfrc*, and *Fth* in EE mice. Genetic blockade of the intestinal *HIF2α*-iron metabolism pathway decreased iron availability in HCT116 cells during hypoxia. The *HIF2α* inhibitor PT2385 suppressed intestinal *HIF2α* expression, decreased iron hypermetabolism, and reduced excessive erythrocytosis in mice. These data support the hypothesis that exposure to high-altitude hypoxia can lead to iron hypermetabolism by activating intestinal *HIF2α* transcriptional regulation, and reduced iron availability improves EE by inhibiting intestinal *HIF2α* signaling.

## 1. Introduction

High-altitude hypoxia can induce excessive erythrocytosis (EE) [1,2]. EE is thought to be a sign of inadequate high-altitude acclimatization [3,4,5], and is one of the critical traits of chronic mountain sickness (CMS) or Monge’s disease in high-altitude populations worldwide [6,7,8]. This excessive pathobiological response has deleterious effects; it results in high hemoglobin (Hb) levels, causing severe hypoxemia and clinical symptoms such as headaches, breathlessness, fatigue, and sleep disorders [9,10,11]. Moreover, the exacerbated erythropoietic response has been suggested to be caused by the high-altitude hypoxia-induced systemic physiological changes in respiratory, cardiovascular, and hormonal responses [12,13,14]. However, the pathophysiological mechanism that results in EE remains under debate.

EE is an uncommon disorder that is marked by an abnormally increased red cell mass and abnormal changes in the body’s physiology. These changes affect the balance between iron availability and the physiology of hypoxic responses, which are important for maintaining oxygen homeostasis [15]. Hypoxia and iron are functionally related; hypoxia stimulates erythropoiesis, which increases the body’s ability to transfer oxygen, and the boost in red blood cells requires a large amount of extra iron, which must be obtained through dietary iron absorption as well as mobilization from iron stores [16]. Over the past decade, research has revealed that the liver-derived hormone hepcidin regulates the major organs of iron metabolism, and intestinal iron absorption is required to sustain systemic iron homeostasis [17]. However, a definite physiological connection between EE and iron metabolism remains unclear.

Hypoxia-inducible factors (*HIFs*) are transcription factors that bind to specific DNA regions known as hypoxia responsive elements (HREs) to initiate homeostatic gene transcription involved in iron metabolism and oxygen availability [18]. *HIFs* regulate many genes that are involved in erythropoiesis and iron metabolism, which are required for tissue oxygen delivery [19]. *HIF2α* activity increases with low oxygen levels in small intestinal enterocytes, activating dietary iron absorption and iron release into the circulation [20]. *HIF2α* plays a key role in regulating cellular iron and oxygen levels, which activates iron transporters at the apical side of the enterocyte and iron exporters at the basolateral side, releasing iron into the circulation and maintaining systemic iron levels [21,22]. *HIF2α* also regulates dietary iron hyperabsorption, which leads to disorders of systemic iron overload, such as β-thalassemia illness [23,24]. However, the precise molecular cues by which intestinal *HIF2α* responds to both erythropoietic requirements and systemic iron metabolism during hypoxia are unknown. Furthermore, it is unknown whether there is a coordinated molecular integration of the intestinal *HIF2α* signaling pathway for EE via the dysregulation of iron metabolism caused by high-altitude hypoxia.

In this study, we hypothesized that high-altitude hypoxia activates intestinal *HIF2α* signaling, thereby increasing iron availability, and inducing EE in high-altitude mice. We examined whether pharmacological or genetic blockade of the intestinal *HIF2α*–iron gene pathway may alleviate iron hypermetabolism in vivo and in vitro and ameliorate EE.

## 2. Materials and Methods

### 2.1. Animals and Treatments

**Figure 1:**
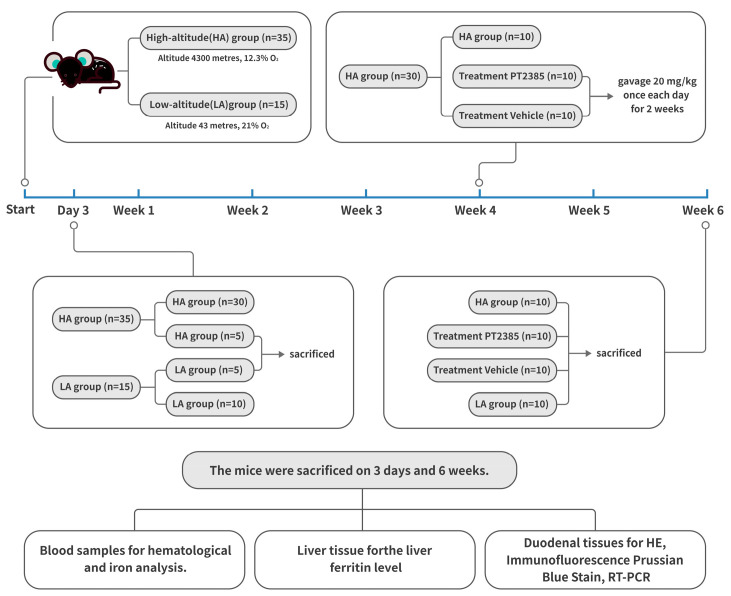
Flow chart of the mouse experimental design.

All mice-related operations described in this study were authorized by the experimental committee of Qinghai University and followed the Chinese Ministry of Health’s animal management policies. The mice used in our experiments were all 6- to 8-week-old male C57BL/6J mice obtained from the Vital River Laboratory Animal Technology Company (Beijing, China). The 50 mice were randomized into four groups. For the low-altitude (LA) group (*n* = 15), mice were housed at an elevation of 43 m and 21% O_2_ (Beijing, China) for six weeks. The high-altitude mice were housed at an elevation of 4300 m, 12.3% O_2_ (Maduo, China) for six weeks. The high-altitude hypoxia mice were divided into three groups: the high-altitude (HA) group (*n* = 15), the PT2385 treatment group (*n* = 10), and the blank drug control (vehicle) group (*n* = 10). For the *HIF2α* inhibitor studies, mice were administered and oral gavage at 20 mg/kg of body weight of either PT2385 (HY12867 MedChem Express, South Brunswick Township, NJ, USA) suspended in saline with 10% DMSO, 40% PEG300, 5% Tween 80, and 45% saline, or a vehicle (10% DMSO, 40% PEG300, 5% Tween 80, and 45% saline) as previously described [25]. Before the treatments, there were no differences in body weight between the mice. All mice were given standard chow and were exposed to a 12-h light-dark cycle. The mouse experimental design is shown in Figure 1.

### 2.2. Cell Culture and Transfection

**Figure 2:**
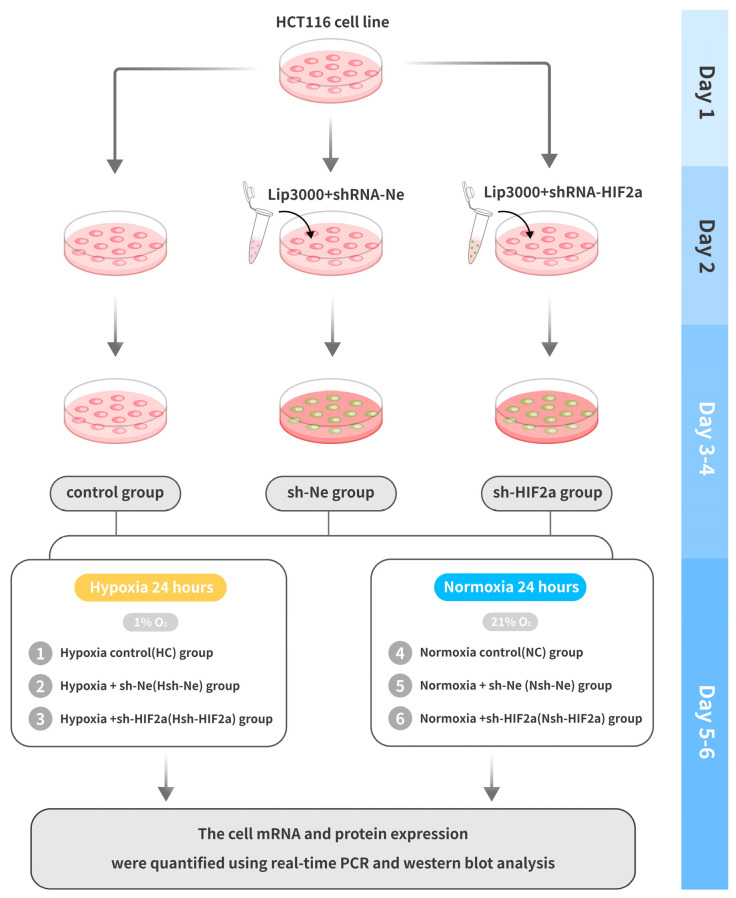
Flow chart of the cell experimental design.

The human intestinal cell line (HCT116) was obtained from the National Infrastructure of Cell Lines (Beijing, China). All cells were maintained in IMDM (Thermo Fisher Scientific, Waltham, MA, USA) with 10% fetal bovine serum (GIBCO, Thermo Fisher Scientific) and 1% penicillin/streptomycin (Solarbio, Beijing, China) in 5% CO_2_ and 21% O_2_ at 37 °C. The short hairpin RNAs targeting human *HIF2α* (sh-*HIF2a*) were designed and produced by Genechem (Shanghai, China). The *HIF2a*-RNAi targeting sequences were cgACCTGAAGATTGAAGTGAT (only the sense strand is shown). The Lipofectamine^TM^ 3000 transfection reagent (L3000001 Thermo Fisher Scientific) was used to transfect sh-*HIF2a* into HCT116 cells. The shRNA negative control (sh-Ne) was used and delivered into cells using the same conditions. After successful cell transfection, the cells were inoculated at a density of 1 × 10^6^/mL (2 mL/well) and cultured in 6-well plates. A total of five passages were performed, and hypoxic and normoxic control studies were performed for each passage. To perform hypoxic studies, cells were grown for 24 h at 37 °C in 1% O_2_ and 5% CO_2_ with balanced N2 [26].The experiment cells were divided into six groups: 1. Hypoxia control (HC) group, cells were grown with 1% O_2_; 2. Hypoxia + sh-Ne (Hsh-Ne) group, cells were transfected with negative control shRNA; 3. Hypoxia +sh-*HIF2a* (Hsh-*HIF2a*) group, cells were transfected with shRNA *HIF2α*; 4. Normoxia control (NC) group, normal oxygen partial pressure (21% O_2_) treatment; 5. Normoxia + sh-Ne (Nsh-Ne) group; 6. Normoxia + sh-*HIF2a* (Nsh-*HIF2a*) group. The successfully generated HCT118 cell knockdown clone’s *mRNA* and protein expression were quantified using real-time PCR and western blot analysis, respectively. The cell experimental design is shown in Figure 2.

### 2.3. Hematological and Iron Analysis

Mice were anaesthetized by injecting urethane (100 mg/kg) into their abdomens. Blood samples were collected for hematological and iron analysis by the retroorbital venous plexus exsanguination. Hemoglobin (Hb), hematocrit (Hct), and red blood counts (RBC) were analyzed using a Mindray Biomedical Electronics BC-5000Vet blood cell analyzer (Shenzhen, China). A Total Iron-Binding Capacity (TIBC) and a Serum Iron Assay Kit (ab239715; Abcam, Cambridge, UK) were used to measure serum iron and serum transferrin saturation (TS). The liver ferritin level was determined via a mouse ferritin enzyme-linked immunosorbent assay kit (E-EL-M0491c; Elabscience, Wuhan, China).

### 2.4. Histology, Immunofluorescence Staining, and Tissue Iron Staining

Fresh duodenal tissues were preserved in 4% paraformaldehyde and encapsulated in paraffin. Hematoxylin-Eosin (HE) Stain Kit was used on duodenal section tissues (G1120; Solarbio). After xylene deparaffinization (3 times, 5min for each), ethanol was used to clean the sections of tissues (wash for 3 min each with 100% ethanol, 95% ethanol, 80% ethanol, and 75% ethanol). Sections (5 µm) were stained with hematoxylin solution for 5 min, rinsed with water, stained for 5 min with eosin solution, and then rinsed with water again. For immunofluorescence staining, duodenal frozen tissues were sectioned (5 µm) and fixed for 15 min in 4% paraformaldehyde. Sections were blocked in a 10% goat serum for 60 min at room temperature before being probed with the polyclonal rabbit anti-HIF2α (1:100 ab199; Abcam) overnight at 4 °C and subsequently with rhodamine-labeled goat anti-rabbit IgG (1:100, ZF-0316; ZSGB-BIO, Beijing, China) for 120 min. After that, a counterstain with DAPI for 5 min. Tissue iron staining was performed with a Prussian Blue Stain (ab150674; Abcam). After deparaffinization in xylene, slides were incubated in a working Iron Stain solution for 3 min, rinsed in water, stained in a nuclear fast red solution for 5 min, rinsed 4 times in water, dehydrated in 95% alcohol, and mounted as directed by the manufacturer.

### 2.5. Real-Time Quantitative PCR

Mouse duodenal tissues and human intestinal cell lines were used to extract total RNA using an RNA Easy Fast Tissue/Cell Kit (DP451; TIANGEN Biotech Co., Beijing, China). The cDNA was constructed utilizing the FastKing gDNA Dispelling RT SuperMix (KR118; TIANGEN) and the QuantStudio5 PCR System was used to amplify genes. The cycling conditions used a 20 μL total reaction volume, and the qPCR process was as follows: pre-amplification at 95 °C for 15 min, followed by 40 cycles of amplification at 95 °C for 10 s, and elongation at 60 °C for 32 s. The fold-change in the genes was measured using the ∆∆Ct method with β-actin as the housekeeping gene. The primers are listed in Table 1.

### 2.6. Western Blot Analysis

RIPA buffer was applied to create whole-cell lysates from human intestinal cell lines (R0010; Solarbio). Total protein content was measured by Pierce™ BCA Protein Assay Kit (23227, Thermo Fisher Scientific). The protein lysates containing 30–40 µg of per well were loaded onto 10% SDS-PAGE, and the separated protein bands were transferred onto a 0.22-µm PVDF membrane and blocked with 5% BSA Blocker solution for 2 h at room temperature. The primary antibodies targeted HIF2α (1:500, ab199; Abcam), FTH (1:1000, ab183781; Abcam), FPN (1:1000, A14884; ABclonal, Wuhan, China), DMT1 (1:1000, ab262715; Abcam), DCYTB (1:1000, ab66048; Abcam), and ACTIN (1:1000, ab8226; Abcam). The secondary antibodies were anti-Rabbit/Mouse from ABclonal. Proteins on the blots were detected using chemiluminescence. ImageJ (version 1.49, Bethesda, MD, USA) was used to calculate protein levels.

### 2.7. Statistical Analysis

Statistical tests and graphs were performed using SPSS 27.0 (IBM Corp., Armonk, NY, USA) and GraphPad Prism 8.0 (GraphPad Software LLC, San Diego, CA, USA). Data are expressed as mean ± standard deviation. The two-tailed unpaired *t*-test was used for comparisons between two groups, whereas a one-way analysis of variance (ANOVA) was used to conduct multi-group analyses. Statistical significance is indicated as * *p* < 0.05.

## 3. Results

### 3.1. EE in High-Altitude Hypoxia Mice Is Accompanied by Enhanced Iron Availability

**Figure 3:**
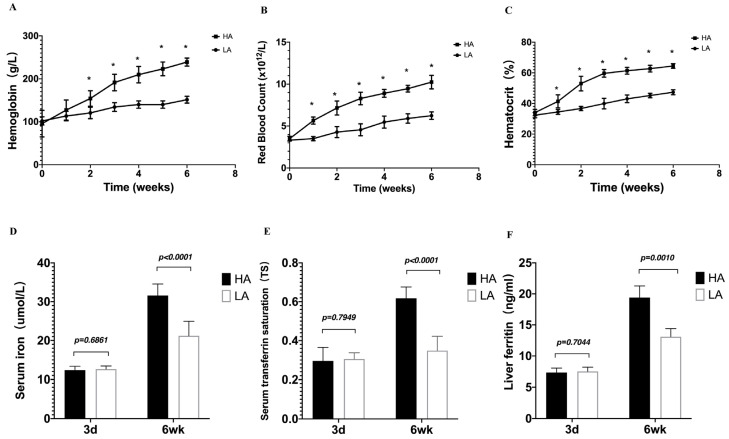
High-altitude hypoxia affects hematological parameters and iron status indicators in mice. (**A**) Hemoglobin levels, (**B**) red blood cell counts, (**C**) hematocrit levels, (**D**) serum iron, (**E**) serum transferrin saturation, and (**F**) liver ferritin. (**A**–**C**) *n* = 10 per group; (**D**–**F**) *n* = 5–10 per group. Data are presented as the mean ± SD. * p < 0.05. Significance was calculated by a two-tailed, unpaired *t*-test.

To understand the physiological connection between EE and iron availability during the high-altitude hypoxia, the mice were observed for 6 weeks, and no deaths occurred during the high-altitude hypoxia exposure period. Hb levels, RBC counts, and Hct in HA mice were notably higher than those in LA mice in a time-dependent manner (Figure 3A–C). Serum iron, serum TS, and liver ferritin reflect the capacity for iron absorption, transportation, and storage. We detected serum iron, TS, and liver ferritin increases at 6 weeks after exposure to high-altitude hypoxia. Compared to the LA group, the iron status of the HA group was significantly increased (Figure 3D–F). These findings suggest that high-altitude hypoxia mice with EE have increased iron availability.

### 3.2. Intestinal HIF2α Signaling Is Associated with Iron Regulation in High-Altitude EE Mice

**Figure 4:**
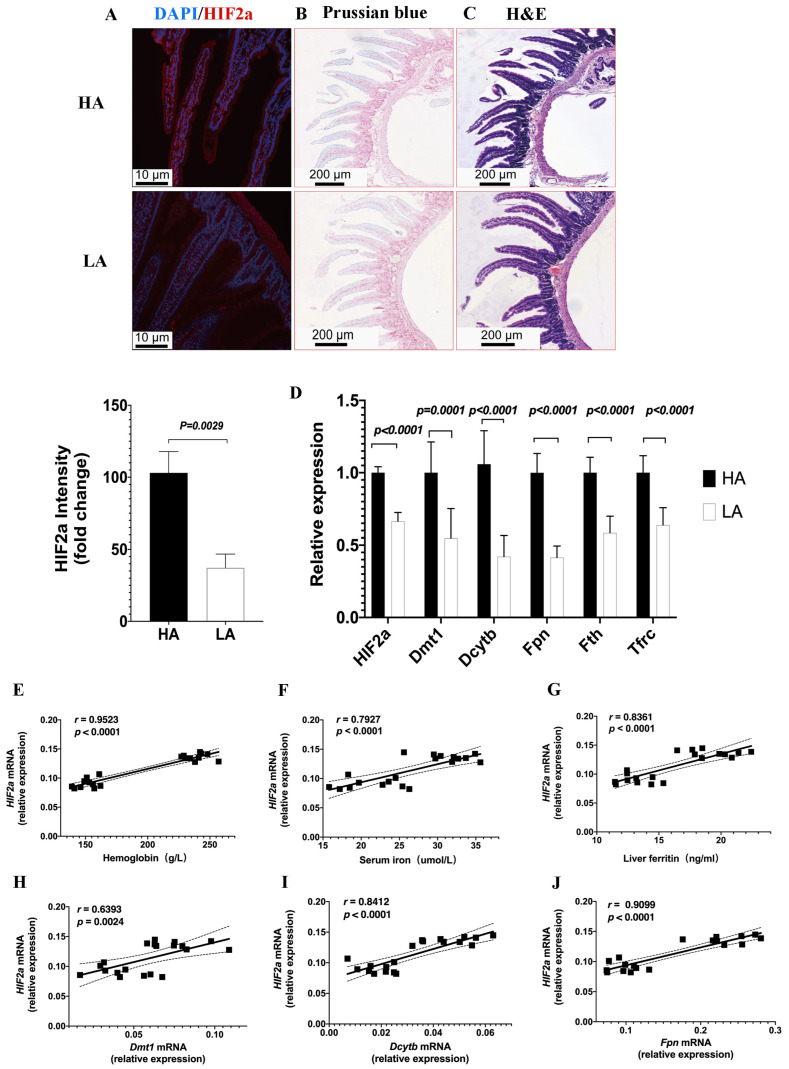
High-altitude hypoxia increases intestinal *HIF2α* levels and is associated with Hb and iron metabolism. (**A**) Immunofluorescence staining of *HIF2α* expression on duodenal sections from the HA and LA groups. Original magnification was ×200. Quantifications of the fluorescence intensity signals are shown in the bar graphs. (**B**) Prussian blue iron staining of duodenal tissues. (**C**) Hematoxylin and eosin staining of duodenal tissues. (**D**) Quantitative PCR for *HIF2α* and iron metabolism genes: *HIF2α*\*Dmt1*\*Dcytb*\*Fpn*\*Fth*\*Tfrc*. (**E**–**J**) Correlative analyses of intestinal *HIF2α* mRNA levels with Hb (**E**), serum iron (**F**), liver ferritin (**G**), *Dmt1* mRNA (**H**), *Dcytb* mRNA (**I**), and *Fpn* mRNA (**J**). (**A**–**C**) *n* = 3 per group, (**D**) *n* = 10 per group, (**E**–**J**) *n* = 10 per group. Correlations were assessed via Pearson’s test. Data are presented as the mean ± SD. Significance was calculated by a two-tailed, unpaired *t*-test.

To investigate the relationship between intestinal *HIF2α* transcriptional expression and iron availability in high-altitude hypoxia, we assessed *HIF2α* expression on duodenal sections from the HA group using immunofluorescence staining (Figure 4A), which revealed significantly higher *HIF2α* expression in the HA group compared to the LA group, suggesting that high-altitude hypoxia responses may trigger intestinal *HIF2α* expression. To confirm whether iron and hypoxia are functionally linked, Prussian blue staining was performed which revealed enhanced progressive iron acquisition in duodenal sections of the high-altitude EE mice compared to the low-altitude mice (Figure 4B). The Hematoxylin and Eosin staining revealed no structural variations between the HA and LA groups (Figure 4C). Moreover, the iron-absorptive and iron-mobilization genes, divalent metal transporter (*Dmt1*), duodenal cytochrome b (*Dcytb*), ferroportin (*Fpn*), and ferritin (*Fth*), as well as transferrin receptor (*Tfrc*), were also markedly upregulated in high-altitude EE mice (Figure 4D). The expression of the intestinal *HIF2α* mRNA was positively associated with Hb, serum iron, and liver ferritin levels, as well as the *Dmt1* mRNA, *Dcytb* mRNA, and *Fpn* mRNA expression levels (Figure 4E–J). These data indicated that activated HIF2α transcriptional signaling was present in the intestines of high-altitude EE mice, along with increased expression of iron metabolism genes.

### 3.3. Knockdown of HIF2a Downregulates Genes Related to Iron Metabolism in HCT116 Cells under Hypoxic Conditions

**Figure 5:**
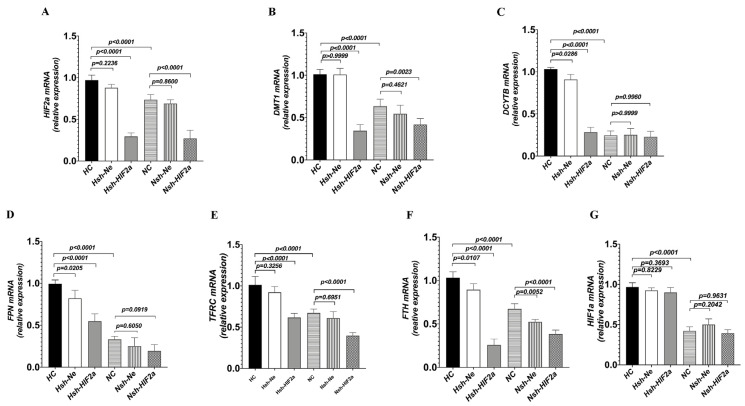
Knockdown of *HIF2α* expression in HCT116 cells decreased iron metabolism gene expression under hypoxic conditions. The cells were transduced with sh-*HIF2a*, as a control sh-Ne to silence the *HIF2α* gene, and then incubated under hypoxic or normoxic conditions for 24 h. (**A**) Quantitative PCR showed the efficiency of *HIF-2α* silencing. (**B**–**F**) Quantitative PCR showed the efficacy of *HIF2α* silencing on *DMT1*, *DCYTB*, *FPN*, *TFRC*, and *FTH*, as well as (**G**) *HIF1a* expression. (**A**) *n* = 5; (**B**–**G**) *n* = 5. Statistical significance was calculated by one-way analysis of variance with Tukey’s post hoc test.

**Figure 6:**
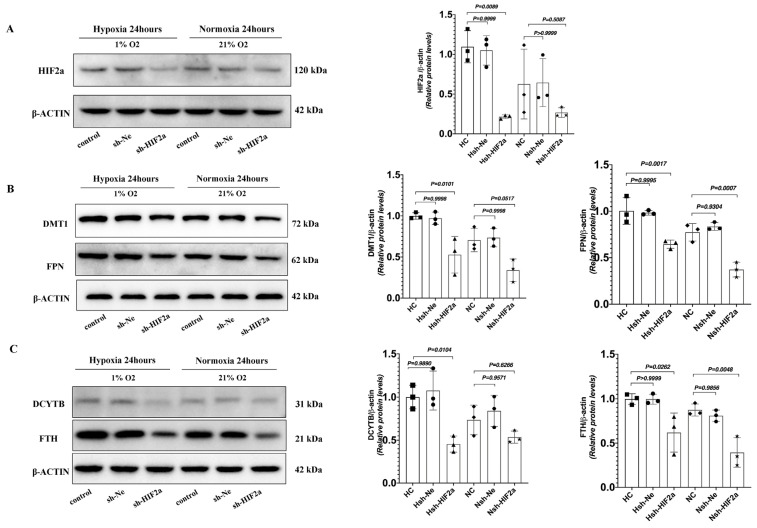
Western Blot analysis of sh-*HIF2a* in HCT116 cells decreased iron metabolism genes protein expression under hypoxic conditions. (**A**) The protein expression of *HIF2a* was determined by Western blot in HCT116 cells. (**B**) The protein expression of *DMT1* and *FPN* was determined by Western blot in HCT116cells. (**C**) The protein expression of *DCYTB* and *FTH* was determined by Western blot in HCT116cells. (**A**–**C**) *n* = 3 per group. Statistical significance was calculated by one-way analysis of variance with Tukey’s post hoc test.

To definitively establish the connection between intestinal *HIF2α* transcriptional activity and iron metabolism in hypoxia, we used short hairpin RNAs (shRNAs) to generate stable knockdowns of *HIF2α mRNA* expression in HCT116 cells (sh-*HIF2a*) (Figure 5A). Compared to shRNA negative control cells (sh-Ne), sh-*HIF2a* significantly decreased the expression of the iron metabolism genes *DMT1, DCYTB, FPN, TFRC,* and *FTH* mRNA (Figure 5B–F) under hypoxia; however, it did not affect the expression of *HIF-1a* mRNA (Figure 5G). Furthermore, sh-*HIF2a* significantly decreased hypoxia-mediated induction of *HIF2a* and iron metabolism genes *DMT1*, *FPN*, *DCYTB*, and *FTH* protein expression levels in the intestinal cell line (Figure 6A–C). These results further support that intestinal *HIF2α* signals can promote iron metabolism and increase iron availability during hypoxia.

### 3.4. Inhibition of Intestinal HIF2α Transcription Decreases Iron Metabolism Gene Expression and Improves Excessive Erythrocytosis in High-Altitude Mice

**Figure 7:**
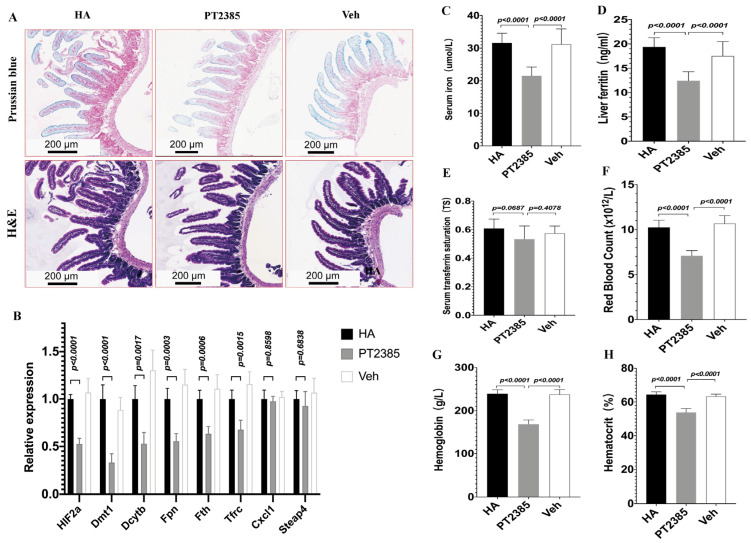
Inhibition of intestinal *HIF2α* using PT2385 decreases iron availability and ameliorates excessive erythrocytosis in high-altitude mice. (**A**) Prussian blue iron staining, Hematoxylin and eosin staining of the double tissue. (**B**) Quantitative PCR analysis of *HIF2α* mRNA and related genes *Dmt1*\*Dcytb*\*Fpn*\*Fth*\*Tfrc*\*Cxcl1*\*Steap4*. (**C**) Serum iron, (**D**) liver ferritin, and (**E**) serum transferrin saturation. (**F**–**H**) Hematological analyses (RBC, Hb, and Hct). (**A**) *n* = 3 per group; (**B**) *n* = 5 per group. (**C**–**H**) *n* = 10 per group. Data are presented as the mean ± SD. Statistical significance was calculated by one-way analysis of variance with Tukey’s post hoc test.

To explore whether decreased iron availability in high-altitude mice ameliorated EE by inhibiting intestinal *HIF2α* transcription, we designed a controlled experiment in HA mice. Since LA mice did not have a high-altitude erythrocytosis phenotype, we treated HA mice with either the PT2385 drug or a blank drug (vehicle) for two weeks. Notably, Prussian blue staining revealed that in progressive duodenal sections, the iron acquisition rate was reduced in PT2385 mice compared to the untreated HA and vehicle mice. Histological examination revealed no major morphological changes between PT2385 and vehicle mice (Figure 7A). Moreover, the PT2385 treatment significantly decreased the levels of mRNA for *HIF2α*, *Dmt1*, *Dcytb*, *Fpn*, *Tfrc*, and *Fth* compared to those of the untreated HA and Veh groups, but not the *HIF2α*-specific inflammatory transcripts *Cxcl1\Steap4* (Figure 7B). Additionally, our qualitative analysis showed that PT2385 mice exhibited lower serum iron and liver ferritin compared to the untreated HA and vehicle mice. However, TS was not different between the *HIF-2α* inhibitor PT2385 mice and vehicle mice (Figure 7C–E); Furthermore, RBC numbers significantly increased in the untreated HA and vehicle mice, as did Hb and Hct levels, and in PT2385 mice, these increases were reduced (Figure 7F–H). These data show that decreased iron availability in high-altitude mice ameliorated EE by inhibiting intestinal *HIF2α* transcription.

## 4. Discussion

Transcription factors play essential roles in human physiology and disease, highlighting the importance of the continued efforts to understand and regulate the expression of the genome in physiological and pathological processes [27]. In this study, we demonstrated that intestinal *HIF2α* is critical for activating a transcriptional program that may increase iron availability through the upregulation of iron metabolism genes expression, and promote EE, providing a potential strategy for the treatment of EE diseases.

Dietary iron intake and body iron stores increase with long-term exposure to high-altitude hypoxia [28]. In the present research, we revealed that excessive erythropoiesis is related to enhanced iron intake and utilization in high-altitude mice. A similar pattern of results was reported previously. Populations living at 2210 m altitude had notably higher levels of hemoglobin, hematocrit, and serum ferritin than their peers living at sea level [29]. Moreover, according to a recent study, Hb and ferritin levels rise significantly every 300 m of residential altitude, starting at 300 m above sea level [20]. In another study, high-altitude migrants had greater serum TS levels than sea-level residents, indicating that more iron was prepared due to increased erythropoietic needs [30]. Consistent with these results, in the present study, we observed increased serum iron levels, TS levels, and liver ferritin levels in high-altitude EE mice, owing to enhanced iron availability from external absorption and intrinsic reuse [31].

High-altitude hypoxia may cause EE. In native Tibetans, a strongly selected *HIF2α* mutation was found to be related to normal hemoglobin at high altitude [32,33]. Similar to this research, we found a significantly higher intestinal *HIF2α* expression in high-altitude EE mice, which suggests that high-altitude hypoxia responses might trigger intestinal *HIF2α* transcriptional expression. *HIF2α* plays an important role in iron hyperabsorption in primary and secondary hemochromatosis [23,24,34]. The activation of *HIF2α* in the intestine promotes dietary iron intake and iron release from storage organs [15]. Based on the findings, the present study raises the hypothesis that intestinal *HIF2α* plays an important role in the regulation of systemic iron homeostasis in high-altitude EE mice, which is supported by the close relationship between *HIF2α* mRNA and protein expression patterns and iron metabolism genes. We also found that intestinal *HIF2α* transcriptional signaling was positively correlated with Hb and iron availability in high-altitude EE mice, and that activated intestinal *HIF2α* resulted in upregulated iron metabolism genes, which can cause an excess of iron supply. Furthermore, the easy access to iron provided by active intestinal *HIF2α* is a risk factor for stimulating EE [28]. Our study indicated that intestinal *HIF2α* transcription is important in the regulation of systemic iron homeostasis and that activating *HIF2α* transcription in the intestines of the high-altitude EE mice resulted in increased iron acquisition, utilization, and storage.

Hypoxic-stimulated erythropoiesis requires the availability of extra iron for heme production [19]. *HIF2α* is essential and sufficient to drive the adaptive increase in iron uptake under hypoxia and erythropoiesis demand by directly transcriptionally activating iron absorption pathways [26,35,36]. In the present study, we evaluated the degree to which intestinal *HIF2α* contributed to the hypoxic modulation of iron metabolism genes. These included the genes implicated in iron-absorptive and iron-transport genes, *Dmt1*, *Dcytb*, *Fpn* and *Tfrc*, which were also markedly upregulated in high-altitude EE mice [37]. We validated this finding in vitro by treating HCT116 cells with *HIF2α*^shRNA^, confirming that *HIF-2α*, but not *HIF-1α*, was a critical regulator of the iron metabolism pathway by measuring the gene and protein expression of *DMT1*, *DCYTB*, *FPN*, and *FTH* in HCT116 cells during hypoxia. We revealed that the intestinal *HIF2α* signal centrally influences the activity of the iron metabolism genes in vivo and in vitro.

High-altitude polycythemia (HAPC) patients currently rely on phlebotomy to reduce erythrocyte levels. However, chronic phlebotomy has side effects such as fatigue that can make patients less likely to stick with their treatment plan [11]. According to recent reports, PT2385 selectively suppresses *HIF2α* transcriptional activity by allosteric regulation, inhibiting *HIF2α* heterodimerization and its transcriptional dimerization partner ARNT/HIF1β [38,39,40]. In this study, we demonstrated that inhibiting intestinal *HIF2α* transcriptional activity with PT2385 prevents and improves abnormal iron absorption and metabolism in high-altitude EE mice, followed by a reduction in serum iron and ferritin levels. However, TS did not differ between the *HIF2α* inhibitor mice and vehicle mice, suggesting a contribution of recycled and stored iron by macrophages of the reticuloendothelial system [41,42]. Reticuloendothelial macrophages act as iron storage and recycle components that are not affected by the *HIF2α* inhibitor [43]. Recent studies have demonstrated associations between *HIF2α* downregulation and blood-related phenotypes [44]. Notably, our findings show that hematologic parameters (Hb, RBC, and Hct) greatly increased in an *HIF2a*-dependent manner during high-altitude hypoxia, which was reversed by treatment with PT2385. The oxygen sensing pathway in duodenal cells was demonstrated to be independent of an individual’s Hb content [20]. Accordingly, with low oxygenation, *HIF2α* activity increased in duodenal enterocytes, activating dietary iron intake and utilization in circulation. Our findings provide compelling support for the use of *HIF2α* inhibitors in the treatment of high-altitude hypoxia with EE diseases, many of which are characterized by intestinal *HIF2α* dysfunction and iron hypermetabolism.

In conclusion, we used a high-altitude EE mouse model to show that intestinal *HIF2α* contributes to iron hyperabsorption and may cause EE. Moreover, we showed that in high-altitude EE mice, decreasing iron availability ameliorates EE by inhibiting intestinal *HIF2α* transcription. We discussed that therapeutic intervention in intestinal *HIF2α* activity could help reduce iron hypermetabolism and improve EE in high-altitude hypoxia. However, animal models do not fully replicate the extent of the EE phenotype seen in humans. Therefore, more preclinical research is needed to determine whether molecules targeting *HIF2α* treatment are beneficial in patients.

## Figures and Tables

**Table 1:** Primers and base sequences for RT-PCR detection.

Species	Gene	Forward Primer	Reverse Primer
*Mouse*	β *-actin*	CTACCTCATGAAGATCCTGACC	CACAGCTTCTCTTTGATGTCAC
	*HIF-2a*	GAGAACCTGACTCTCAAAAACG	GTTGTTGTAGACTCTCACTTGC
	*Dmt1*	TTTTGGACAAATATGGCTTGCG	TACTCATATCCAAACGTGAGGG
	*Dcytb*	GTGTTTGAGTACCACAATGTCC	TGGAAGCAGAAAGACGAAAAAG
	*Fpn*	TTGTGTGTGATCTCCGTATTCA	GTTGTAAAGACGGTCTCAGGTA
	*Tfrc*	TCACACTCTCTCAGCTTTAGTG	TGGTTTCTGAAGAGGGTTTCAT
	*Fth*	TAAAGAAACCAGACCGTGATGA	ATTCACACTCTTTTCCAAGTGC
	*Cxcl1*	GGCTGGGATTCACCTCAAGAACATC	TGAGTGTGGCTATGACTTCGGTTTG
	*Steap4*	CCTCTGTGCTGTGCGTCTTCTTC	ACACGATTCGGGATGGAAATGGC
*Human*	β *-ACTIN*	GGCACCACACCTTCTACAATGAGC	GATAGCACAGCCTGGATAGCAACG
	*HIF-1a*	CCATTAGAAAGCAGTTCCGCAAGC	GTGGTAGTGGTGGCATTAGCAGTAG
	*HIF-2a*	ATCAGCAAGTTCATGGGACTTA	AAACCAGAGCCATTTTTGAGAC
	*DMT1*	CATCCTCACATTTACGAGCTTG	CCAACCCAAGTAGAACACAAAG
	*DCYTB*	GCCAGAAGGTGTTTTCGTAAAT	TGGTAGAATTTGGCTCCTTAGG
	*FPN*	ACAATACGAAGGATTGACCAGT	ATACCAAGTTCCATCCCGAAAT
	*TFRC*	TGAACCAATACAGAGCAGACAT	GTTTTCTCAGCATTCCCGAAAT
	*FTH*	CTCCTACGTTTACCTGTCCATG	CAAGTCATCAGGCACATACAAG

## Data Availability

Data are contained within the article.

## Abbreviations

CMS	Chronic Mountain sickness
EE	Excessive erythrocytosis
HA	High-altitude
HC	Hypoxia control
*HIF*	Hypoxia-inducible factors
*DMT1*	Divalent metal transporter
*DCYTB*	Duodenal cytochrome b
*FPN*	Ferroportin
*TFRC*	Transferrin receptor
*FTH*	Ferritin
*CXCL1*	C-X-C motif chemokine ligand 1
*STEAP4*	STEAP family member 4
LA	Low-altitude
NC	Normoxia control
RBC	Red blood cells
Hb	Hemoglobin
Hct	Hematocrit
TIBC	Total Iron Binding Capacity
TS	Transferrin saturation
